# Survival Models on Unobserved Heterogeneity and their Applications in Analyzing Large-scale Survey Data

**DOI:** 10.4172/2155-6180.1000191

**Published:** 2014-04-30

**Authors:** Xian Liu

**Affiliations:** 1 DoD Deployment Health Clinical Center, Walter Reed National Military Medical Center, Bethesda, MD 20889, USA; 2 Department of Psychiatry, F. Edward Hebert School of Medicine, Uniformed Services University of the Health Sciences, Bethesda, MD 20814, USA

**Keywords:** Clusters, Correlated data, Hazard rate models, Survival analysis, Unobserved, Heterogeneity

## Abstract

In survival analysis, researchers often encounter multivariate survival time data, in which failure times are correlated even in the presence of model covariates. It is argued that because observations are clustered by unobserved heterogeneity, the application of standard survival models can result in biased parameter estimates and erroneous model-based predictions. In this article, the author describes and compares four methods handling unobserved heterogeneity in survival analysis: the Andersen-Gill approach, the robust sandwich variance estimator, the hazard model with individual frailty, and the retransformation method. An empirical analysis provides strong evidence that in the presence of strong unobserved heterogeneity, the application of a standard survival model can yield equally robust parameter estimates and the likelihood ratio statistic as does a corresponding model adding an additional parameter for random effects. When predicting the survival function, however, a standard model on multivariate survival time data can result in serious prediction bias. The retransformation method is effective to derive an adjustment factor for correctly predicting the survival function.

## Introduction

Most regression models are performed by assuming conditional independence of observations in the presence of specified model parameters. With regard to survival data, an individual’s event time *T* is generally assumed to follow a univariate distribution, with heterogeneity of event occurrences primarily accounted for by the effect of a number of measurable individual or contextual factors [1–6]. In certain circumstances, however, failure times are correlated even in the presence of specified model parameters, so that the independence hypothesis can be violated. Such internally clustered survival data are generally referred to as survival time data with unobserved heterogeneity. Empirical examples of the multivariate survival data structure include repeated events [1,7], twin survival patterns [8,9], the occurrence of vision loss in the left and right eyes [10], parental history of a given disease on the incidence in the offspring [11], and mortality of married couples at older ages [12]. Because observations may be clustered by unobserved heterogeneity, the application of standard survival models can result in biased parameter estimates and an erroneous model-based prediction.

Over the years, statisticians and other quantitative methodologists have developed a variety of statistical models to handle survival time data with unobserved heterogeneity. Nevertheless, there is a lack of consensus regarding how to apply these survival models in different situations. Most of the survival models on unobserved heterogeneity are built upon biomedical settings characterized by the randomized clinical trial design with small sample size or by a lack of measureable predictors on survival processes. As a result, these methodologically advanced techniques are relatively unfamiliar to researchers of other disciplines who base their analyses on observational data. For example, given the complexity of social events, social scientists often perform regression analysis in the context of a conceptual model, specifying various causal associations as guided by existing theories or specific research interests. Large-scale survey data are regularly collected and used in empirical analyses to ensure the statistical power in constructing and estimating a complex causal model. Given such a unique perspective, techniques borrowed from biostatistics must be verified, evaluated, and modified before they can be utilized effectively in those applied sciences. The statistical models handling survival data with unobserved heterogeneity are no exception.

In this article, four families of regression modeling on survival time data with unobserved heterogeneity are described and compared: the Andersen and Gill [13] approach, the robust sandwich variance estimator [14], the hazard model with individual frailty [15,16], and the retransformation method [17]. Other survival models in this regard are more or less the extensions of the above four families. The focus of this study is placed upon the applicability of these survival models in analyzing large-scale survey data. For this reason, an empirical illustration is provided on the application of the aforementioned survival models, using data of a large-scale longitudinal, multidisciplinary, and U.S. population-based survey.

## Multivariate Survival and Hazard Functions

In the multivariate survival data, lifetime variables *T*_1_, *T*_2_, …․, *T_q_* are associated within a specific level-2 unit *i* with *q* ≥ 2. According to probability theory, the multivariate cumulative distribution function (c.d.f.) over a series of time (*t*_1_, …․, *t_q_*), denoted by *F*(*t*_1_, …․, *t_q_*), is

(1) $$S(t_{1,},\ldots ․,t_{q})=\text{Pr}(T_{1}>t_{1},\ldots ․,T_{q}>t_{q}).$$

Defined as the probability that no event occurs from time 0 to time series (*t*_1_, …․, *t_q_*), the multivariate survival function, denoted by *S*(*t*_1_, …․, *t_q_*), is

(2) $$S(t_{1,},\ldots ․,t_{q})=\text{Pr}(T_{1}>t_{1},\ldots ․,T_{q}>t_{q}).$$

It may be mentioned that when *q*>1, *F* and *S* are no longer complementary of each other.

Likewise, the multivariate density function, given its intimate association with the survival function, is given by

(3) $$f(t_{1},\ldots ․t_{q})=-\frac{\partial^{q}S(t_{1},\ldots ․,t_{q})}{\partial t_{1}\ldots \partial t_{q}}.$$

The marginal hazard functions at time *t* for *q* members of a given cluster, denoted by {λ_1_(*t*), …․, *λ_q_*(*t*)}, are given by

(4) $$\lambda_{1}(t)=-\frac{\partial S(t_{1},\ldots ․,t_{q})/\partial t_{1}}{S(t_{1},\ldots ․,t_{q})}{|}_{t_{1}=\ldots ․=t_{q}=t},\ldots \lambda_{q}(t)=-\frac{\partial S(t_{1},\ldots ․,t_{q})/\partial t_{q}}{S(t_{1},\ldots ․,t_{q})}{|}_{t_{1}=\ldots ․=t_{q}=t}.$$

The hazard rates for the members of a cluster are associated because they share some common characteristic. Therefore, it is plausible to assume that for those clustered members, one’s failure time predicts that of another, and so is the reverse. Given this assumption, a common random effect within a cluster, regularly denoted by a multiplicative term θ, can be specified to indicate the extent to which {*T*_1_, …․, *T_q_*} are associated. Varying over clusters, θ is a random effect between clusters, but it is fixed within a cluster. If θ=1 within a cluster, the marginal hazard rates {λ_1_(*t*), …․, *λ_q_*(*t*)} are independent of each other thus suggesting the absence of an association between {*T*_1_, …․, *T_q_*}. If θ>1, the failure times for the q members are positively associated; and neglect of this term can lead to biased parameter estimates on the hazard rate due to the violation of the independence hypothesis. When the condition θ>1 is considered in a statistical model, observations within the cluster are thought to be conditionally independent, so that more efficient parameter estimates can be derived. The scenario θ<1 is a condition that rarely occurs in reality, so that it is not discussed in this study.

Assuming that all members of a given cluster share the same θ, then this within-cluster fixed effect can be factored out from the specification of multivariate processes. For example, the multivariate survival function can be mathematically written by

(5) $$S(t_{1,}\ldots ․,t_{q})=\theta \int_{t_{1}}^{\infty }f(u_{1},\ldots ,t_{q})du_{1}\ldots ․\int_{t_{q}}^{\infty }f(t_{1},\ldots ․,u_{q})du_{q}.$$

In Equation (5), the joint survival function is simply expressed as the product of *q* marginal survival functions multiplied by θ. As a consequence, the correlation of any two survival times within the cluster is under control if θ is known.

In the multivariate regression analysis, much of the between-clusters random effect θ is statistically addressed by specifying theoretically related covariates. More technically, θ can be parameterized by a parameter vector θ. If the elements specified in θ can fundamentally explain the association among survival times {*T*_1_, …․, *T_q_*}, there is no sufficient space remaining for supporting further parameterization in residuals. Consequently, the baseline hazard rates are conditionally independent thus can be viewed as following a univariate distribution. Many researchers contend, however, that the hazard rates for some members may be associated because they share some genetically common but unobservable characteristic. If observed covariates do not considerably reflect the information of such unobserved factors, the parameter estimates can be statistically inefficient and inconsistent.

## Andersen-Gill Model

Given flexibility of the counting process formulation and the powerful martingale theory [1,4], counting processes are used in much of the literature of statistical modeling on survival time data with unobserved heterogeneity. In this system, the hazard function for a level-1 unit *j* at time *t*, given an underlying hazard function λ_0_, is

(6) $$\lambda_{j}(t)=\lambda_{0}(t)\text{exp}(Z_{j}^{\prime }\beta ),$$

where **β** is a *M*×1 vector of regression parameters, and the covariate vector ***Z**_j_* represents covariates for observation *j*.

Andersen and Gill [13] consider it statistical feasible under certain conditions to extend the above specification to the regression analysis on the intensity of a repeated event, which follows a multivariate distribution of T within an individual. From parameter estimates obtained from maximizing the complete or the partial likelihood function, the integrated intensity processes, denoted by Λ(*t*) in counting processes, can be obtained by a linear interpolation between failures times:

(7) $$\mathrm{\Lambda ̂}(t)=\sum_{T_{l}\leq t}[\frac{\delta_{j}}{\sum_{l\in ℛ(t_{j})}\text{exp}(Z_{l}\beta )}].$$

where *δ_j_* is the 0/1 (1=event, 0=censored) censoring indicator and *R*(*t_i_*) is the risk set at a specific survival time.

Equation (7) is flexible to permit multiple events up to time *t* as its computation is not restricted to a single event. Let *N_i_* ≡ {*N_i_* (*t*),*t* ≥ 0}be the number of observed events experienced over time *t* for individual *i* with *N_i_* (0) = 0 and the sample paths, or trajectory, of the counting process be right continuous step functions. Then, as an alternative definition in terms of counting processes, a joint likelihood function *L*(**β**, Λ) is proposed to model the intensity rate given the past event history:

(8) $$\lambda_{Z}(t)dt=E\{dN(t)|{ℱ}_{t-}\}=\lambda_{0}(t)\text{exp}[Z_{i}^{\prime }(t)\beta_{0}]dt,$$

where **β**_0_ is the true parameter vector. The term *F_t−._* is the σ-algebra generated from the process history prior to *t* about survival, censoring, and covariates; substantively, it indicates what has been experienced in terms of counting processes before a given observed time. The covariate vector ***Z***(t) is defined as time-dependent for reflecting the influences of previous events on future recurrences. So, in the context of repeated events, an individual is a cluster and $Z_{i}^{\prime }(t)$ represents $Z_{j}^{\prime }$.

If ***Z***(*t*) reflects information of clustering, the regression coefficient **β** can be flexibly estimated over the multivariate survival data of repeated events. Let the count *Y* denote the number at risk just at *t* for failing in an infinitesimal interval (*t*, *t*+d*t*), and $\mathrm{N̅}=\sum_{i=1}^{n}N_{i}$ is the risk set for the occurrence of each jump (the first and the recurrent events). Then, the partial likelihood for *n* independent triples {*N_i_*, *Y_i_*, ***Z**_i_* (*t*)}, where *i*=1,…․,*n*, is analogous to the standard Cox formulation but using a different terminological system. After some algebra, the log partial likelihood function can be written by

(9) $$\text{Log}L_{p}(\beta )=\sum_{i=1}^{n}\int_{0}^{t}Z_{i}^{\prime }(u)\beta dN_{i}(u)-\int_{0}^{t}\text{log}\{\sum_{i=1}^{n}Y_{i}(u)\text{exp}[Z_{i}^{\prime }(u)\beta ]\}d\mathrm{N̅}(u),$$

Given score function ***Ũ***(**β**) = (∂log*L_p_*/∂β_1_,…․,∂log*L_p_*/∂*β_M_*), the total score statistic at time *t* is

(10) $$Ũ(\beta ,t)=\sum_{i=1}^{n}\int_{0}^{t}\{Z_{i}(u)-\mathrm{Z̅}(\beta ,u)\}dN_{i}(u),$$

where the second term within the brace represents the expected covariate vector over a given risk set:

$${\mathrm{Z̅}}_{i}(\beta ,u)=\frac{\sum_{i=1}^{n}Y_{i}(u)Z_{i}(u)\text{exp}[Z_{i}^{\prime }(u)\beta ]}{\sum_{i=1}^{n}Y_{i}(u)\text{exp}[Z_{i}^{\prime }(u)\beta ]}.$$

In the martingale theory, d*N*(t) is a submartingale [1,4], and therefore, the score function with respect to **β**_0_ can be expressed in terms of a function of martingales:

(11) $$U(\beta ,t)=\sum \int \{Z_{i}(u)-\mathrm{Z̅}(\beta ,u)\}dM_{i}(u),$$

where

$$M_{i}(t)=N_{i}(t)-\int_{0}^{t}Y_{i}(u)\text{exp}[Z_{i}^{\prime }(u)\beta_{0}]d\Lambda_{0}(u)$$

is the difference over (0, *t*) between the observed number of events for individual *i* and the expected value of the cumulative intensity rate derived from a regression model.

Given Equation (11), the score function can be conveniently expressed in terms of a permutation test based on residuals computed for the regression on covariates, rather than on the regression coefficients [18]. The {*M*_1_(․),…․,*M_n_*(․)} series are defined as martingales if the *M_i_*(*t*) increment in any *t* is independent and if any two *M_i_*, *M_i_*’ at any *t*, where *i*≠*i’*, are uncorrelated with each other. A differentiated martingale, in many ways, behaves like an ordinary residual of linear regression models with mean 0 and without autocorrelation [1]. Statisticians have developed the martingale central limit theorem for mathematically proving the stochastic property in martingales [1,4,19]. While mathematically complex in its derivation, the martingale central limit theorem literally states that when jumps of a martingale converge to a multivariate normal distribution, its trajectory tends to an asymptotically transformed Wiener process with mean 0 and variance-covariance ***V***(*t*). Therefore, in the Andersen-Gill model the correlation of repeated events is reflected in ***Z***(*t*), and consequently, {*M*_1_(․),…․,*M_n_*(․)} series are conditionally independent.

Provided that {*M*_1_(․),…․,*M_n_*(․)} are martingales, the Fisher information matrix, denoted by ***I***(***β**,t*) and defined as the minus second partial derivatives of the log partial likelihood function with respect to **β**, can be readily derived by using the standard procedure. Andersen and Gill [13] proved that statistically, the process $\sqrt{n}(\mathrm{\beta ̂}-\beta_{0})$ tends to converge in probability to a normal vector with mean **0** and the covariance matrix ***I***^−1^ (**β̂**,*t*) for large samples. If this inference holds, the score, the Wald, and the likelihood ratio test statistics are well defined under the null hypothesis that all regression coefficients be zero.

The Andersen-Gill model stipulates that the large sample behavior follows if the correlation of repeated events is reflected in covariates and the semi-parametric baseline hazard function. The specification of time-dependent covariates or theory-based interaction terms can considerably mitigate correlation of clustered data thereby making potential dependence of residuals insignificant. Technically, the Andersen-Gill intensity rate model does not differ significantly from a standard Cox model [20]. Therefore, if its underlying hypothesis holds, the Andersen-Gill approach on repeated events can be applied to all types of survival data with unobserved heterogeneity, as long as the specified fixed effects and the underlying hazard function reflect much of unobserved heterogeneity.

## The Robust Sandwich Variance Estimator

According to Andersen and Gill [13], the observed Fisher information matrix given **β̂**, denoted by ***I***(**β̂**,*t*), tends to converge in probability to the nonsingular deterministic matrix. Given this statistical property, the asymptotic limit of (**β̂**−**β**) is 0 as long as censoring is independent of ***Z*** [21]. Therefore, the point estimates of regression coefficients in the Andersen-Gill approach or the standard Cox model are asymptotically unbiased, even with the existence of strong unobserved heterogeneity in failure times [22].

Wei, Lin, and Weissfeld [14] contend, however, the joint distribution of the score function $\sqrt{n}{Ũ}_{1}(\beta_{1},\infty ),\ldots ․,\sqrt{n}{Ũ}_{q}(\beta_{q},\infty )$ cannot be statistically viewed as local martingales if correlation among failure times exists. Because of the condition cov(*M_j_*,*M*_*j*_′) ≠ 0 in the presence of unobserved heterogeneity, where *j* ≠ *j*′, the martingale central limit theorem does not apply to the series {*M*_1_,…,*M_q_*}. As a result, the inverse of the observed information matrix ***Î***^−1^ (**β̂**) does not provide an adequate variance estimator of **β̂**. A robust covariance matrix for **β̂** is thus needed to account for covariance in $\sqrt{n}{Ũ}_{1}(\beta_{1},\infty ),\ldots ․,\sqrt{n}{Ũ}_{q}(\beta_{q},\infty )$ for satisfying the condition $\sqrt{n}{Ũ}_{k}(\beta_{k},\infty )\to N[0,V(\beta_{k})]$. Accordingly, WLW have developed the robust sandwich variance estimator for addressing unobserved heterogeneity in correlated survival data.

The robust sandwich variance method does not specify the pattern of dependence among correlated failure times, either; rather, it constructs a robust variance-covariance estimator externally to account for the within-clusters covariance thus yielding consistent and asymptotically normal parameter estimates. Let

(12) $$\mathrm{\Sigma ̂}(\mathrm{\beta ̂})=n^{-1}\sum_{i=1}^{n}{Ũ}_{i}(\mathrm{\beta ̂}){Ũ}_{i}(\mathrm{\beta ̂})\prime ,$$

where, in the context of repeated events, *i* represents cluster *i* (*i*=1, …․, *n*), **Σ̂** is the score statistic variance estimator, and ***Ũ**_i_* (**β**) consists of {***Ũ***_*i*1_ (**β**),…․,***Ũ**_iq_* (**β**)}. Then the asymptotic covariance matrix of the estimated regression coefficients is given by

(13) $$\mathrm{V̂}(\mathrm{\beta ̂})={Î}^{-1}(\mathrm{\beta ̂})\mathrm{\Sigma ̂}(\mathrm{\beta ̂}){Î}^{-1}(\mathrm{\beta ̂}).$$

Equation (13) is the so-called “sandwich” variance estimator. As a result of such an adjustment, the random vector $\sqrt{n}(\mathrm{\beta ̂}-\beta_{0})$ is asymptotically normal with mean **0** and a covariance matrix that can be estimated by ***V̂*** (**β̂**) [21], from which the valid Wald score can be derived for testing the null hypothesis on **β̂**. As both **β̂** and ***I*** (**β̂**) can be obtained from the standard Cox model, this robust variance estimator does not involve additional statistical inference as long as the underlying clustering factor can be identified. Therefore, the robust “sandwich” variance estimator is an external method only dealing with the standard error estimation, not for the parameter estimation. Given its attachment to the standard Cox model and flexibility to adjust for the biased variance matrix, this “sandwich” approach has tremendous appeal in biostatistics [5]. When the Andersen-Gill model is correctly specified, however, correlation among failure times is much mitigated thus leading to the condition ***V̂*** (**β̂**) ≈ ***Î***^−1^ (**β̂**). If this approximation holds, the use of the sandwich variance estimator becomes unnecessary.

## Hazard Models with Individual Frailty

Some methodologists attempt to analyze correlated survival data by defining a quantity termed “frailty,” a convenient notion used most frequently by mathematical biologists and demographers [16,23]. Here, frailty may refer to a broad range of dimensions, such as genetic predisposition, physiological senescence parameters, economic capability, family history of disease, and the like [24,25]. In the frailty theory, individuals in a random sample have different levels of frailty, and the frailer ones tend to die sooner than do the others. Consequently, the existence of an unobserved “frailty” effect can alter patterns of general mortality and mortality differences. There are numerous reports in recent years that age-specific mortality rates of different subpopulations cross in ways that are unanticipated [26–28]; biological explanations of this phenomenon are based on the frailty theory assuming the intersecting mortality functions to represent heterogeneous populations that differ in frailty [16,29].

The original frailty model assumes a heterogeneous lifetime pattern to address the latent frailty effect [15,16,23]. Specifically, the unobserved frailty factor can be represented by an unobservable random effect, which impacts the baseline hazard function multiplicatively:

(14) $$\lambda_{i}(t)=z_{i}\lambda_{0}(t)\text{exp}[Z_{i}^{\prime }(t)\beta ],$$

where *z_i_* is the frailty score for individual *i* (individual here is the level-1 unit), with values varying around the grand mean of this variable. Hence, the frailty model specified by Equation (14) is basically a random-effects proportional hazard model. This model has tremendous appeal because the hazard function with frailty is expressed as the regular proportional hazard model plus a multiplier representing the random disturbances. Given the nonnegative nature of the hazard rate, an individual *z* is also nonnegative, presumed to follow a given parametric distribution. Conditional on the random effect *z* and other specified parameters, survival times are assumed to be independent of each other, so that more efficient estimates of **β** can be derived.

The distribution of the random effect *z* determines how a frailty factor affects the value of the hazard rate. Vaupel et al. [16] recommend that given its flexible mathematical properties, a gamma distribution should be used to reflect frailty at birth, with parameters η and ν, where η>0 is the scale parameter and ν>0 is the shape parameter. The mean and variance of the gamma distribution are well defined, given by

(15a) $$E(z)=\frac{\nu }{\eta },$$

(15b) $$\text{var}(z)=\frac{\nu }{\eta^{2}}.$$

Equation (14) specifies a conditional hazard model as it parameterizes the conditional distribution of *T* given and the observed covariates. As the frailty effect is unobserved, researchers generally desire estimates of covariates’ effects on the hazard rate of specific subgroups. Therefore, it is perhaps more appropriate to specify the gamma-distributed frailty model in terms of the marginal mean, given by

(16) $$E[\lambda_{i}(t,z,Z_{i})]=\int z\lambda_{0}(t)\text{exp}[Z_{i}^{\prime }(t)\beta ]dF(z)=\frac{\lambda_{0}(t)\text{exp}[Z_{i}^{\prime }(t)\beta ]\nu }{\eta }=\lambda_{0}(t)\text{exp}[Z_{i}^{\prime }(t)\beta +\text{log}(\frac{\nu }{\eta })],$$

where the error distributional function *F* is the cumulative density function.

If both gamma parameters are free, the frailty model is not identifiable [1]; therefore the mean of *z*’s must be set at one by imposing the conditions that η=ν and var(*z*)=1/ν=1/η. There are a variety of Bayes-type techniques for approximating the integral of the likelihood over the frailty effect, such as the expectation-maximization estimator [30] and the Gaussian quadrature algorithm [31]. While these estimators sometimes yield different parameter estimates on the hazard function, in non-pathological cases such differences are generally negligible.

Some scientists [12,15,24] comment on the use of a gamma distribution for the frailty factor *z*. Despite its mathematical attractiveness given its simple densities, the gamma distribution has some specification weaknesses [15,18]. Aalen [15] suggests that the nature of the frailty effect must be derived from biological knowledge and theoretical assumptions about the known risk factors the frailty factor represents. He provides an example in terms of the effect of blood pressure and serum cholesterol on the incidence of myocardial infarction among middle aged men, contending that both factors are approximately normally distributed with some skewness to the right. It follows that the relative risk due to these factors should be approximately lognormally distributed.

Taking log values on both sides of equation (14) gives rise to

$$\text{log}[\lambda_{i}(t)]=\text{log}[\lambda_{0}(t)]+Z_{i}^{\prime }(t)\beta +\text{log}(z_{i})=\text{log}[\lambda_{0}(t)]+Z_{i}^{\prime }(t)\beta +\varepsilon_{i},$$

where log[λ_0_(*t)*] can be viewed as the intercept in the linear predictor, and *ε_i_* is log(*z_i_*). If *z* is lognormally distributed, then ε is normally distributed with mean 0 and variance σ^2^, thereby leading to a typical generalized linear regression model with a normally distributed error term.

Given a transformed error term with normal distribution, the mean and variance of the frailty factor *z* are

(18) $$E[\text{exp}(\varepsilon )]=\text{exp}(\varepsilon +\frac{\sigma^{2}}{2})=\text{exp}(\frac{\sigma^{2}}{2}),$$

and

(19) $$\text{var}[\text{exp}(\varepsilon )]=\text{exp}(2\varepsilon )\{\text{exp}[2\sigma^{2}-\text{exp}(\sigma^{2})]\}=\text{exp}[2\sigma^{2}-\text{exp}(\sigma^{2})].$$

Obviously, the lognormal specification for the frailty factor is a more convenient, parsimonious choice than a gamma distribution if the lognormal distribution of the random term is reasonably assumed. In the frailty theory, however, the assertion of lognormality is considered empirically too strong given the impact of the “survival of the fittest” process.

The various frailty models have proven an efficient, valid statistical perspective to handle unobserved heterogeneity in many occasions. Sometimes, the applications of frailty models may encounter certain specification problems in analyzing large-scale survey data [18]. The multiplicative effect of the unobserved frailty factor, independently drawn from a parametric distribution, is assumed to be orthogonal to inferences about other parameters. In reality, an individual’s frailty should be closely interacted with observed factors, and some researchers even use an observable covariate as the proxy for measuring an individual’s level of frailty [32]. When the risk set at a given observed failure time contains a decent number of individuals with covariates scaled at many levels, unobserved heterogeneity from the latent “frailty” factor can be considerably reflected in the fixed effects and the underlying hazard function thereby making the addition of a random term statistically redundant [2,33]. Given these arguments, research into large sample behavior of frailty models is ongoing.

## Retransformation Method

Although parameterization of random effects does not necessarily impacts the estimation of model parameters, it does not mean that the standard proportional hazard model yields robust and consistent estimators on nonlinear predictions given the existence of unobserved heterogeneity. Random disturbances cannot be overlooked in predicting the hazard or the survival function. First, we cannot assume that the error term is zero for each observation, which implies an exact linear dependence of unobserved heterogeneity on the covariate vector ***Z***. Second, it is equally misleading to assume that the expected value of the error term in a linear predictor is zero when predicting the hazard rate. Consider, for example, the case of a lognormal distribution: when one retransforms a normal distribution of random errors to a lognormally distributed function, the expected value of the multiplicative random effect is not unity given the properties of a lognormal distribution. Even if the true parameters **β** are known, the function λ_0_(*t*)exp(***Z***′**β**) is not the correct estimate of E[λ(*t*)], such that

$$E[\lambda (t)]=\lambda_{0}(t)\text{exp}[Z\prime (t)\beta ]E[\text{exp}(\varepsilon )]\neq \lambda_{0}(t)\text{exp}[Z\prime (t)\beta ].$$

Equations (18) and (19) specify basic properties of a lognormal distribution. In those equations, σ^2^ is the mean square error; and its mode, median and moments all have the same functional form [34]. Whereas the median of exp(ε) is simply exp[E(ε)] that implies a multiplicative effect of one, the positive skewness of the lognormal distribution mandates that the median lies below the mean, with equality holding if and only if σ^2^ = 0. Thus, neglect of retransforming the error term in estimating any log-linear equation with a reduced form leads to a median function, rather than a mean function. Consequently, unbiased and consistent quantities on the log(hazard) cannot be retransformed into unbiased and consistent quantities on the hazard rate without considering retransformation of random components.

Provided that ε is normally distributed and uncorrelated with λ_0_(*t*) and ***Z***, the expected value of the hazard rate given unobserved heterogeneity can be written by

(20) $$E\{\lambda (t)|Z(t)\}=\lambda_{0}(t)\text{exp}[Z_{i}^{\prime }(t)\beta +\frac{\sigma^{2}}{2}]=\lambda_{0}(t)\text{exp}[Z_{i}^{\prime }(t)\beta ]\text{exp}(\frac{\sigma^{2}}{2})=\lambda_{0}(t)\text{exp}[Z_{i}^{\prime }(t)\beta ]\Phi ,$$

where Φ=exp(σ^2^/2) is an adjustment factor in the mean for the retransformation in the hazard function assuming ε to be normally distributed with mean zero and variance σ^2^.

While the hazard rate is unobservable, the variance of random errors can be either derived from a likelihood function integrated over the individual-level random effect or estimated externally. In the literature of generalized linear mixed models, there is a variety of approximation methods proposed to derive Bayes-typed parameter estimators given random effects [35–37]. Though generally working well in longitudinal data analysis, these integration techniques are often found ineffective in analyzing large-scale survival data given the specification of an underlying stochastic process in *T* [2,38]. Thus, the external perspective seems to be a more appropriate choice for nonlinear predictions. Among the external approaches in this area, the most popular method is perhaps the “threshold concept” approach [39]. In the logistic regression, random errors are assumed to follow a standard logistic distribution with mean 0 and variance π^2^/3, while for a probit regression random errors are assumed to follow a standard normal distribution with mean 0 and variance 1. This approach, however, is not practicable for survival models because all hazard models, no matter how many covariates are considered, would have exactly the same variance of random disturbances. As McCullagh and Nelder [40] comment, the assumption of a continuous latent distribution is a rough model requirement, though providing a useful concept for generalized linear modeling.

Another convenient approach to obtain the expected value of random errors uses the empirical data. Specifically, the researcher can recognize the predicted hazard rates obtained from a “full” model as an unbiased and consistent set of *λ_i_* to calculate *s*^2^, a sample estimate for σ^2^. If random errors in a well-specified full model are truly ignorable after appropriate justification, Φ can be approximated from the standard formula on variance between the full model and a corresponding reduced-form equation [17]. In performing this approach, a fraction of random disturbances from omitting one or more significant predictors may be absorbed into the intercept. As a result, the intercept needs to be fully specified in the calculation of Φ. Consequently, the Cox model and the partial likelihood estimator are inappropriate for the application of this method.

When the assumption of normality for ε cannot be satisfied, the factor [exp(σ^2^/2)] is not the correct adjustment in the mean for retransformation from the logarithmic scale to the untransformed hazard rate, so that this estimator can lead to incorrect nonlinear predictions of the hazard rate. In this situation, Duan’s [41] smearing estimate can be applied.

First, assuming the vector ***Z*** to have full rank and ε is not normally distributed, we have

(21) $$E\{\lambda (t)|Z(t)\}=E\{\lambda_{0}(t)\text{exp}[Z\prime (t)\beta +\varepsilon ]\}=\int \lambda_{0}(t)\text{exp}[Z\prime (t)\beta +\varepsilon ]dF(\Psi ),$$

where Ψ is the multiplicative random effect exp(ε).

When the error distribution function *F* is unknown, this cumulative density function *F* can be replaced by its empirical estimate *F̂_n_*, referred to as the smearing estimate [41]:

(22) $$E\{\lambda (t)|Z(t)\}=\int \lambda_{0}(t)\text{exp}[Z\prime (t)\beta +\varepsilon ]d{\mathrm{F̂}}_{n}(\Psi )=\frac{1}{n}\sum_{i=1}^{n}\lambda_{0}(t)\text{exp}[Z\prime (t)\beta +{\mathrm{\varepsilon ̂}}_{i}]=[{\mathrm{\lambda ̂}}_{0}(t)\text{exp}[Z\prime (t)\mathrm{\beta ̂}]]n^{-1}\sum_{i=1}^{n}\text{exp}({\mathrm{\varepsilon ̂}}_{i}),$$

where **β̂** can be obtained by employing the maximum likelihood procedure without considering unobserved heterogeneity given the martingale central limit theorem [7,13,14].

A consistent estimate of the hazard rate given covariate vector ***Z*** can be obtained by

(23) $$E\{\lambda (t)|Z(t)\}={\mathrm{\lambda ̂}}_{0}(t)\text{exp}[Z\prime (t)\mathrm{\beta ̂}]\mathrm{\Psi ̂},$$

where

(24) $$\mathrm{\Psi ̂}=\frac{\sum_{i=1}^{n}e^{\text{log}\mathrm{\lambda ̂}(t|Z_{i})-\text{log}\mathrm{\lambda ̂}(t|Z_{i},Y_{i})}}{n-1}=\frac{\sum_{i=1}^{n}\text{exp}[Z_{i}(\mathrm{\beta ̂}-\mathrm{\alpha ̂})-Y_{i}^{\prime }\mathrm{\gamma ̂}]}{n-1}.$$

where ***Y*** is the vector of covariates incorporated in the full model but not in the reduced-form model, **α** is the vector of regression coefficients for ***Z*** in the full model, and **γ** is the vector of regression coefficients for ***Y*** in the full hazard model. Analytically, Equations (23) and (24) are meant to estimate an unknown error distribution by the empirical c.d.f. of the estimated regression residuals. This “smearing” effect does not completely eliminate bias when the true distribution of random errors is unknown; but the overall prediction bias is negligible if a large sample is used to find a nonparametric distribution. This nonparametric retransformation method predicts the longitudinal health data accurately [38,42].

## Illustration

In this illustration, an empirical example is provided on mortality differences between American older veterans and nonveterans. The observation range is a four–five year interval from 1993/94 to the end of 1997. Given a single data set, the applicability of various survival models on unobserved heterogeneity is assessed and examined.

### Data

The data used for the analysis come from the Survey of Asset and Health Dynamics among the Oldest Old (AHEAD). This longitudinal survey is a nationally representative investigation of older Americans conducted by the Institute for Social Research (ISR), University of Michigan, as a supplement to the Health and Retirement Study. The Wave I of the AHEAD survey was conducted between October 1993 and April 1994. A sample of individuals age 70 or older (born in 1923 or earlier) was identified throughout the HRS screening of an area probability sample of households in the nation. This procedure identified 9,473 households and 11,965 individuals in the target area range. The Wave I respondents have been followed by telephone every second or third year, with proxy interviewing designed for those deceased between two successive surveys. By now, AHEAD survey registers nine waves of investigation in 1993, 1995, 1998, 2000, 2002, 2004, 2006, 2008, and 2010. As a longitudinal, multidisciplinary, and U.S. population-based study, AHEAD provides a highly representative and reliable data base for the survival analysis of older Americans age 70 or older. Survival information throughout the follow-up waves has been obtained by a link to the data of National Death Index (NDI). Given the purpose of illustration, I randomly select 2,000 persons from the original AHEAD sample for the analysis.

### Measures

This illustration uses Wave I data (baseline survey) and the survival data in a four-to-five year period. Time of death is recorded for those who died between the Wave I interview and December 31, 1997. Of 2,000 Wave I respondents, 332 were identified dead during the interval. For each of the deceased in this four-to-five year observation period, the duration in months from Wave I interview to the time of death is recorded. Other respondents are right censored.

The AHEAD survey acquires detailed information on a number of domains, including demographic characteristics, health status, health care use, disability, retirement plans, and health and life insurance. Given a relatively short observation period, all covariates considered in this empirical illustration are time-independent variables with their values fixed at Wave I survey. Veteran status, the main explanatory variable in this illustration, is measured as a dichotomous variable (veteran=1, nonveteran=0), named “VET” in the statistical analysis. Over 90 percent of veterans in the dataset served in the military during World War II.

The control variables include age, gender, educational attainment, and marital status. The time-independent assumption on marital status might lead to some bias in estimating its effect. Other explanatory variables, however, are either stable over time (veteran status, gender, and educational attainment) or change simultaneously with time (age) thus not posing any threats to the validity of time-independent hypothesis. In particular, age is defined as the actual years of age reported by respondents at the time of the Wave I survey. As the starting age of the data is 70 years, this variable is rescaled to be centered at age 70 (actual age–70), termed AGE_70 in the analysis. Given the theoretical hypothesis on the trend of mortality convergence and crossover between older veterans and nonveterans, an interaction term is created between Vet and Age_70. Statistically, the specification of this interaction can absorb massive information on heterogeneous selection of survival.

Gender is indexed as a dichotomous variable (women=1, men=0), and educational attainment, an approximate proxy for socioeconomic status, is measured as the total number of years in school, assuming the influence of education on mortality to be a continuous process [43]. Lastly, marital status is specified as a dummy variable, with currently married=1, else=0. For analytic convenience without loss of generality, these three control variables are all rescaled to be centered at sample means, termed Female_cnd, Educ_cnd, and Married_cnd, respectively. Empirically, the mean of a dichotomous variable indicates the likelihood or propensity of being in the group coded 1; it can also be understood as the expected proportion in the population a random sample represents.

The effects of two health factors–physical health conditions and mental disorders–on the hazard function are closely examined in the preliminary data analysis. While their effects on the hazard rate are very strongly statistically significant in the presence of other covariates, in this example these two health factors are purposefully excluded from the final hazard model. Therefore, there is definitely additional clustering in the survival data, even in the presence of the six covariates considered.

Table 1 displays the mean (or proportion), the standard deviation, and the coding scheme of each original covariate and the names of the centered variables.

### Survival models on unobserved heterogeneity

The removal of two statistically significant predictor factors from the hazard model guarantees that survival data in the regression analysis are conditionally dependent. In this illustration, four hazard models are applied–the standard proportional hazard (PH) regression, two frailty models, and the retransformation method–with an attempt to compare their capabilities for addressing unobserved heterogeneity. The robust sandwich variance estimator is not considered in the illustration because, given the present dataset, this method does not modify the variance estimates considerably.

Most regression models handling unobserved heterogeneity are designed by parameterizing random effects in order to accomplish conditional independence among observations. Because the retransformation method requires the specification of a complete likelihood function, the Weibull proportional hazard model is used with the addition of a term for individual-level random effects. The adjusted Weibull model is given by

(25) $$\lambda (t|Z,z)=\lambda_{0}\mathrm{p̃}{\left(\lambda_{0}t\right)}^{\mathrm{p̃}-1}\text{exp}[Z\prime \beta +\text{log}(z)],$$

where, within the construct of the Weibull hazard model, λ_0_ is the scale parameter, *p̃* is the shape parameter, and *z* represents the random effect. The vector ***Z*** in this analysis contains the six covariates mentioned earlier. Empirically, the Weibull hazard model often derives identical estimates of covariates’ regression coefficients as does the Cox model [44], so that the application of the Cox model would generate the same fixed effects. With the addition of a random effect term, the adjusted Weibull hazard function no longer possesses the proportional and monotonic properties. In this illustration, however, the quality of the parameter estimates is the major concern, so is the potential impact of the random effect, so that the shape of a baseline distributional function is not of interest. The arbitrarily assumed random effect is not considered in the standard proportional hazard model assuming unobserved heterogeneity to be statistically accounted for by incorporating theoretically relevant covariates.

The crucial part of creating a frailty model is to select a specific distributional function for the frailty effect *z*. In this analysis, two frailty models are fitted with random effects integrated into the likelihood function: (a) the Weibull hazard model with random variable *z* assumed to be lognormally distributed, and (b) the Weibull hazard model with *z* assumed to have a gamma distribution. In fitting the second frailty model, the mean of *z*’s is set at one given the condition that η=ν and var(*z*)=1/ν, as conventionally applied.

In performing the retransformation method, normality is assumed for the random effects in the linear predictor; and the coefficient Φ is estimated externally thereby not impacting the estimation of other parameters. The full model, used to calculate empirically-based random errors, considers eight covariates–the aforementioned six explanatory variables plus the two omitted health factors.

The operational objective of this illustration is three-fold: first, to examine whether the likelihood function integrated over the random effect is statistically effective in fitting the Weibull hazard model. Here, the model chi-square criterion, given the likelihood ratio statistic, is used to test the null hypothesis that the addition of a random term does not fit the AHEAD survival data significantly better than does the standard PH model. The second objective is to know whether the hazard model with a gamma distributed random effect fits the data significantly better than does the model with a lognormal distribution, also using the model chi-square criterion on the null hypothesis. The last operational objective is to assess whether the retransformation method yields a statistically significant smearing estimate for nonlinear predictions. For strengthening this last procedure, two predicted survival curves are compared for older persons age 85, one generated from the standard Weibull hazard model and one from the retransformation method. Because veteran status is used as a major independent variable, those survival curves are created for older veterans and nonveterans separately. Given an equal sample size for the estimation of each model, the degree of freedom can be well specified, so that these comparisons can be conducted effectively.

The standard Weibull survival function at time *t* is

(26) $$Ŝ(t|\mathrm{p̃},Z)=\text{exp}[-\text{exp}(Z\prime \mathrm{\beta ̂})t^{\hat{\tilde{p}}}].$$

In the retransformation method, the survival function, assuming random effects in the linear predictor to be normally distributed, is given by

(27) $$Ŝ(t|\mathrm{p̃},Z,\mathrm{\Phi ̂})=\text{exp}[-\text{exp}[(Z\prime \mathrm{\beta ̂})+\text{log}(\mathrm{\Phi ̂})]t^{\hat{\tilde{p}}}].$$

All four models are fitted using the SAS PROC NLMIXED procedure. Theoretical implications can be summarized by examining different sets of parameter estimates, the model fit statistics, and the predicted survival functions.

## Results

Table 2 summarizes the results derived from the four hazard rate models on the AHEAD data. The first hazard model in the table is the regression specifying a number of fixed effects without a random term, assuming unobserved heterogeneity to be completely accounted for by the fixed effects. The next two hazard models are the Weibull functions fitted by maximizing an approximation to the likelihood integrated over the unobserved random effect, distributed either as a lognormal or as a gamma function. The fourth model is the Weibull regression with the smearing effect estimated externally to the maximization of the likelihood function.

The estimated regression coefficients in the first two models are almost identical, with trivial differences only in some of the standard error estimates and *p*-values. In both models, the regression coefficient of veteran status is negative, while that of the interaction term between veteran status and age is positive, which, combined, suggest a typical pattern of mortality convergence and crossover between older veterans and nonveterans, and the subsequent excess mortality among older veterans. The effect of veteran status is considered statistically significant given the statistical significance of the interaction term. The variance of the normally distributed random effect, not exactly reported in Table 2, is 0.000006, so that the mean frailty effect with a lognormal distribution is exp(0.000006/2)=1.000003. Obviously, the integration of a lognormally distributed random term to the likelihood function does not impact the estimation of the parameters at all given the specification of a flexible baseline hazard function. More important, the value of −2×(log likelihood ratio), distributed as χ^2^ with 1 degree of freedom on the null hypothesis, remains unchanged after the addition of the random effect, highlighting the statistical redundancy of this arbitrarily assumed random term in this particular data set.

Compared to the first two models, the third Weibull model, assuming gamma distributed random effects, derives different parameter estimates. First, the random effect parameter var(*z*) is statistically significant (0.6421; *t*=10.80, *p*<0.0001). Second, the estimate of the Weibull shape factor is considerably increased, 1.915, compared to 1.265 obtained from the first two models given the specification of a different scale factor. Third, absolute values of the regression coefficients are much elevated, thanks to the increased amount of the shape parameter. Compared to the frailty model with a lognormal distribution, this statistical model generates no gain in the model fit because the difference in the value of −2×(log likelihood) is negative (4246.7 versus 4250.9). According to the statistical criterion that less is better with regard to this statistic, the frailty model with a gamma distribution does not improve the model fit compared to the first two models. Consequently, the null hypothesis that the integration of a random effect in the likelihood does not significantly improve the estimation of the hazard model cannot be rejected. There are some more refined model fit indices for handling complex data structures, such as Akaike’s information criterion (AIC) and Bayesian information criterion (BIC); in the present comparisons, however, using another model fit statistic would probably generate the same conclusion given an equal sample size for these models.

Clearly, the integration of an arbitrarily assumed random term to the likelihood function is not statistically supported in this example, as the flexible, monotonic Weibull baseline function and the fixed effects absorbs substantial information of the random effects. Thus, the retransformation method, an external approach to generate unbiased estimates of functionals of the regression parameters when random effects are present, can serve as an alternative for estimating the random effects. The last two columns of Table 2 display the results of the fourth hazard model. As Φ is estimated empirically, other parameter estimates and the model fit statistic are exactly the same as those estimated for the standard PH model. The value of Φ is 1.271, very strongly statistically significant.

**Figure 1:**
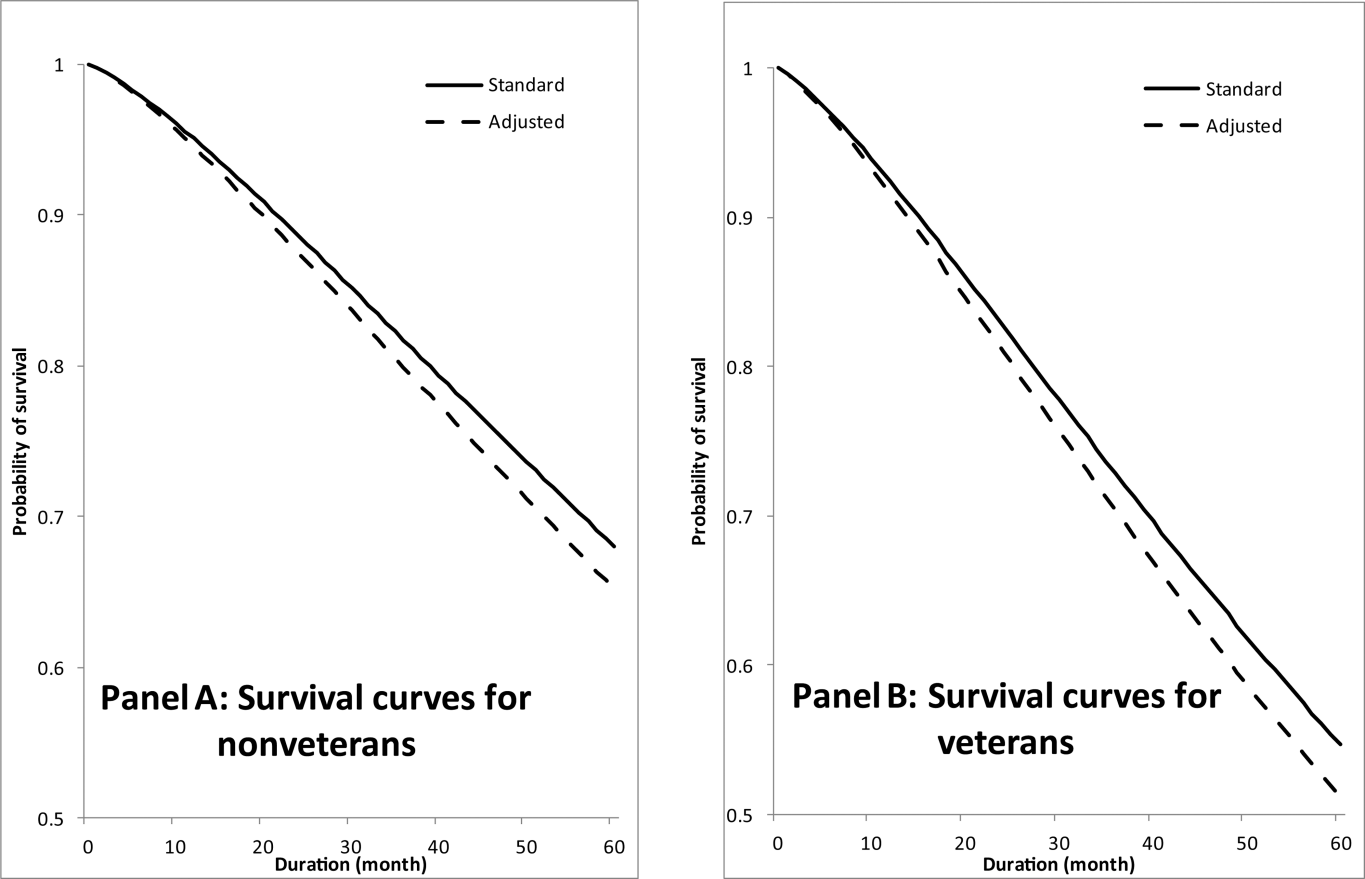
Predicted survival curves for veterans and nonveterans from the standard approach and the retransformation method.

Figure 1 plots the evolution of the survival functions predicted from the standard proportional hazard model and the retransformation method, respectively. In Panel A, which compares the predicted survival curves among older nonveterans, there is a distinct and systematic separation between the two curves. At each time point following the origin of time, the predicted probability of survival obtained from the standard hazard model is considerably higher than from the retransformation method. In Panel B, the two survival curves decline more sharply thereby indicating faster mortality acceleration among older veterans than among nonveterans; however, the separation between the two predicted curves remains the same as in Panel A. The substantive meaningfulness of such separations is governed by the statistical significance of Φ derived from the retransformation method.

## Conclusions

This study displays that parameterization of random effects in the hazard model does not necessarily function effectively for capturing unobserved heterogeneity in analyzing large-scale survey data. According to Andersen and Gill [13], if correlation in survival data is reflected in the covariates, the large sample behavior follows thereby making the parameter estimates asymptotically unbiased. The flexibility of the baseline hazard function in the parametric Weibull or the semi-parametric Cox model can usually mitigate the impact of unobserved heterogeneity and therefore loose the assumption on the frailty term. Occasionally, serious bias in the variance estimator of **β̂** arises while the point estimates are asymptotically unbiased; in such situations, the variance-covariance estimates can be easily adjusted by applying the robust sandwich variance estimator. Even so, the standard PH model can result in serious prediction bias. In the illustration presented in this study, for example, inherent random disturbances exist in survival data due to removal of two theoretically important, statistically significant predictor factors. As the two frailty models are not shown to be effective for capturing the effect of additional clustering, the application of the retransformation method is useful for deriving an adjustment factor for nonlinear predictions of lifetime processes.

It must be emphasized that the results displayed in this study do not suggest that frailty models, particularly the model with a gamma distribution, are not useful. The results presented above just demonstrate that for this particular example with this particular data set, the application of two frailty models does not significantly improve the quality of parameter estimates and the likelihood ratio statistic when random effects are present. Under different patterns regarding unobserved heterogeneity and other factors such as sample size, the performance of the four models may differ significantly. In behavioral science, empirical data often come from large-scale observational surveys, from which a large quantity of variables are available for the specification of complex conceptual frameworks. If a theoretical model is correctly specified for guiding data analysis, the impact of unobserved heterogeneity on parameter estimates can be immensely mitigated by specified fixed effects thereby making additional parameterization redundant [2,13,33,38]. Here, the desirable large-sample behavior is effective because a stochastic time-to-event process, particularly in the Cox model, can largely wash out the impact of unobserved heterogeneity [2]. In these occasions, the incorporation of an additional frailty factor in the PH model is not supported by large-sample theory [18] therefore is misspecified. Such a statistical advantage is usually not pertinent in biomedical studies, which regularly use survival data either of a small sample size or with a lack of measurable variables. In those situations, the frailty theory and its attaching models are highly valuable for addressing unobserved heterogeneity. The example given in this study does not derive general directives by using a single data set, and some other empirical works show that ignoring unobserved heterogeneity can lead to incorrect estimates. Therefore, ignorability of random effects in the survival model must be carefully assessed and justified from situation to situation, using the likelihood ratio test or other more refined statistical criteria. Large scale simulation is needed to investigate general patterns of unobserved heterogeneity under different conditions.

## Figures and Tables

**Table 1:** Mean or proportion, standard deviation, coding scheme of covariates: Older Americans (*n*=2,000).

Explanatory Variable	Mean or proportion	Standard deviation	Coding Scheme	Variable name in analysis
Veteran status (proportion)	0.19	–	1=veteran, 0=nonveteran	Vet
Age (mean)	75.79	6.59	Actual number of years from birth	Age_70
Female (proportion)	0.67	–	1=yes, 0=no	Female_cnd
Education (mean)	11.11	3.55	Actual years attending school	Educ_cnd
Currently married (proportion)	0.55	–	1=yes, 0=no	Married_cnd

Note: In the analysis, age_70=(actual age–70); the rest of the covariates, except vet, are mean-centered variables.

**Table 2:** Results of four hazard rate models on the mortality of older Americans between 1993 and 1997: Fixed-effects and frailty models (*n*=2,000).

Covariate & other	Standard PH model		Lognormal frailty model		Gamma frailty model		Retransformation method	
Statistics	coefficient	Hazard ratio	coefficient	Hazard ratio	coefficient	Hazard ratio	coefficient	Hazard ratio
Veteran status	−0.241	0.786	−0.241	0.786	−0.392	0.676	−0.241	0.786
Age_70	0.064***	1.066	0.064***	1.066	0.101***	1.106	0.064***	1.066
Vet × Age_70	0.046**	1.047	0.046*	1.047	0.082*	1.085	0.046**	1.047
Female_cnd	−0.556***	0.573	−0.556***	0.573	−0.911***	0.402	−0.556***	0.573
Educ_cnd	−0.016	0.985	−0.016	0.985	−0.027	0.973	−0.016	0.985
Married_cnd	−0.168	0.846	−0.168	0.846	−0.286	0.751	−0.168	0.846
Intercept	−7.092***		−7.092***		−11.493***		−7.092***	
Random effect			0.000		0.642***		0.480***	
Mean frailty score			1.000		1.000		1.271	
Shape coefficient	1.265***		1.265***		1.915***		1.265***	
−2 log likelihood	4246.70		4246.70		4250.9		4246.70	

Note: The parameter “shape” is tested by (*p̃* –1.0)/SE.

* 0.05<*p*<0.10;

** 0.01<*p*<0.05;

*** *p*<0.01.
